# Intravenous fentanyl self-administration in male and female C57BL/6J and DBA/2J mice

**DOI:** 10.1038/s41598-023-27992-8

**Published:** 2023-01-16

**Authors:** Michael Leonardo, Sarah Brunty, Jessica Huffman, Alexis Kastigar, Price E. Dickson

**Affiliations:** Department of Biomedical Sciences, Joan C. Edwards School of Medicine, Marshall University, 1700 3rd Ave., Huntington, WV 25703 USA

**Keywords:** Genetics, Behavioural genetics, Neuroscience, Reward, Psychiatric disorders, Addiction

## Abstract

The genetic mechanisms underlying fentanyl addiction, a highly heritable disease, are unknown. Identifying these mechanisms will lead to better risk assessment, early diagnosis, and improved intervention. To this end, we used intravenous fentanyl self-administration to quantify classical self-administration phenotypes and addiction-like fentanyl seeking in male and female mice from the two founder strains of the BXD recombinant inbred mouse panel (C57BL/6J and DBA/2J). We reached three primary conclusions from these experiments. First, mice from all groups rapidly acquired intravenous fentanyl self-administration and exhibited a dose–response curve, extinction burst, and extinction of the learned self-administration response. Second, fentanyl intake (during acquisition and dose response) and fentanyl seeking (during extinction) were equivalent among groups. Third, strain effects, sex effects, or both were identified for several addiction-like behaviors (cue-induced reinstatement, stress-induced reinstatement, escalation of intravenous fentanyl self-administration). Collectively, these data indicate that C57BL/6J and DBA/2J mice of both sexes were able to acquire, regulate, and extinguish intravenous fentanyl self-administration. Moreover, these data reveal novel strain and sex effects on addiction-like behaviors in the context of intravenous fentanyl self-administration in mice and indicate that the full BXD panel can be used to identify and dissect the genetic mechanisms underlying these effects.

## Introduction

In the United States, fentanyl is the leading cause of death for adults aged 18–45^1,2^. This striking statistic underscores the magnitude of the addiction epidemic in the United States and highlights that the principal driver of this growing public health crisis is fentanyl. Indeed, deaths from fentanyl increased 1.7 times from 2020 to 2021, and deaths from fentanyl overdose represent 64% of the ~ 100,000 drug overdose deaths that occurred in the United States during that time^1,2^. Massive efforts over the last several decades to reduce the distribution and use of illicit drugs have failed to curtail the increasing incidence of drug addiction^3^. A likely cause of this is our failure to identify and characterize the underlying genetic mechanism driving addiction which is one of the most heritable psychiatric disorders^4–7^. Identifying the genetic underpinnings of the distinct behavioral phenomena which drive addiction will lead to better risk assessment, early diagnosis, and improved intervention.

Intravenous drug self-administration is widely considered to be the gold standard for preclinical addiction phenotyping^8^. The construct validity of the self-administration approach stems from its volitional nature and the ability to model multiple addiction stages including acquisition of drug use, escalation of drug use, drug-seeking when the drug is not available, and relapse following periods of abstinence^9–16^. Although the use of intravenous self-administration in the mouse has grown over the past 20 years, the technical challenges of microsurgery and maintenance of catheterized mice has limited its use to a small number of laboratories. Moreover, the majority of mouse intravenous self-administration papers report the investigation of cocaine self-administration as opposed to the self-administration of opiates or opioids, and the majority of these studies have been limited to male mice from the C57BL/6J mouse strain. With regard to fentanyl specifically, a search of PubMed for “mouse intravenous self-administration fentanyl” (performed on Oct 6th 2022) failed to return papers reporting the use of intravenous fentanyl self-administration in mice. In summary, little is known about the genetic mechanisms underlying this important addiction phenotype in the mouse.

Systems genetics using experimental mouse populations^17,18^ is an elegant and robust approach for discovery of genetic drivers underlying heritable behaviors such as intravenous drug self-administration. The BXD population, a biparental recombinant inbred mouse panel derived from the C57BL/6J and DBA/2J founder strains, has been used extensively in this context^6,19–25^. As an initial step in this process, the C57BL/6J and DBA/2J founder strains can be assessed for strain differences. If the founder strains differ on a phenotype, this reveals that the full BXD panel can be used to discover the underlying genetic drivers influencing variation on that phenotype. The integration of a systems genetics approach with a construct valid assay for indexing addiction-like behaviors, such as intravenous fentanyl self-administration, provides a strong approach for discovering fundamental and translational addiction mechanisms in mice.

In the present study, we quantified intravenous fentanyl self-administration in male and female mice from the founder strains of the BXD recombinant inbred mouse panel. Adult C57BL/6J and DBA/2J mice of both sexes were implanted with a jugular catheter and, following at least two weeks of recovery, tested on intravenous fentanyl self-administration. In daily sessions over six weeks, we quantified multiple self-administration phenotypes including acquisition, dose–response, escalation, extinction, cue-induced reinstatement, restraint stress-induced reinstatement, and IV fentanyl-induced reinstatement. These data were subsequently analyzed to assess strain differences, sex differences, and strain-by-sex interactions.

## Materials and methods

### Subjects

Experiments were conducted in the Department of Biomedical Sciences within the Joan C. Edwards School of Medicine at Marshall University. Experiments were approved by the Institutional Animal Care and Use Committee at Marshall University and conducted in accordance with the National Institutes of Health Guidelines for the Care and Use of Laboratory Animals and with the ARRIVE guidelines. Efforts were made to reduce the number of animals used and to minimize animal pain and discomfort.

Male and female C57BL/6J and DBA/2J mice were ordered from The Jackson Laboratory (JAX) at 3 weeks of age and were used as experimental subjects (JAX stock numbers 000664, 000671). With the exception of one C57BL/6J female and one DBA/2J female, all mice that began behavioral testing successfully completed all of the intravenous fentanyl self-administration stages shown in Table 1. In total, 32 mice completed behavioral testing and were used in the statistical analyses described below. The 32 mice that completed the experiment were 6 male C57BL/6J mice, 7 female C57BL/6J mice, 12 male DBA/2J mice, and 7 female DBA/2J mice.

**Table 1:** Stages of the intravenous fentanyl self-administration experiment.

Stage	Dose (μg/kg)	Criteria to advance to the next stage
Acquisition	56	8 sessions ≥ 1 infusion per session
Dose–response	18	3 sessions
”	5.6	”
”	1.8	”
”	0.56	”
”	0.18	”
Restabilization	56	”
Extinction	n/a	7 sessions
Cue-induced reinstatement	n/a	1 session
Extinction	n/a	3 sessions
Stress-induced reinstatement	n/a	1 session
Extinction	n/a	3 sessions
Fentanyl-induced reinstatement	280 bolus	1 session

Mice were maintained in a temperature-controlled environment (21 ± 1 °C) on a 12:12 light:dark cycle (lights on at 0600). Mice had free access to food and water throughout the experiment except for the brief time in the testing apparatus. Prior to entering the experiment, mice were group housed with mice from the same sex and same strain. Following jugular catheterization surgery, mice were singly housed for the remainder of the experiment. Mice were housed in cages that contained Nestlets and Shepherd Shacks both prior to the experiment when group housed and during the experiment when singly housed.

### Apparatus

The operant conditioning equipment used in this study has been described in detail previously^26^. Briefly, intravenous fentanyl self-administration data were collected using 32 modular mouse operant conditioning chambers enclosed in sound attenuating cubicles (Med Associates; St. Albans, Vermont). Two retractable response levers were mounted to the right and left sides of the front wall (henceforth active lever and inactive lever, respectively). A stimulus light was mounted directly above each of the two levers. A house light was centrally mounted on the front wall of each chamber. A 25-gauge single-channel plastic swivel was mounted to a counterbalanced lever-arm attached to the lid of the chamber. An infusion pump was mounted within the sound attenuating cubicle outside of the operant conditioning chamber. Tubing was used to connect a 20 mL syringe mounted on the infusion pump to the swivel. During fentanyl self-administration testing, tubing was used to connect the externalized catheter port on the midscapular region of the mouse to the plastic swivel. Operant conditioning chambers were controlled by two Med Associates control units using MED-PC V software.

### Jugular catheterization surgery

At 12 weeks of age, an indwelling catheter was implanted into the right external jugular vein under oxygen/isoflurane anesthesia using our previously described procedures^26^. Briefly, the catheter was inserted 12 mm into the jugular vein and anchored with sutures. The catheter was tunneled subcutaneously to an incision in the midscapular region where it was connected to an externalized catheter access port. Mice were allowed to recover for at least 14 days before behavioral testing began.

### Intravenous fentanyl self-administration

Following at least two weeks of surgical recovery, mice were tested on an intravenous fentanyl self-administration paradigm consisting of several stages (Table 1) during which classical pharmacological measures and addiction-like behaviors were quantified. Methodological details of these stages are described in the subsections below. Throughout the experiment, mice were tested in two-hour sessions at the same time daily seven days per week. Session length was 120 min for all stages with the exception of the stress-induced reinstatement stage for which the length was 60 min; this was to accommodate 60 min of restraint stress which occurred immediately preceding behavioral testing. Throughout the experiment and irrespective of dose, infusion volume was ~ 20 µL and infusion duration was ~ 2.5 s. Infusion duration, and consequently volume, were programmatically adjusted based on mouse weight to hold µg/kg/infusion of fentanyl constant across mice.

Mice were tested in daily session using our previously described methods^26^. Briefly, to maintain patency, catheters were flushed before and after each daily testing session with 20 µL of a heparin lock solution (100 U/mL heparin/saline). To forestall bacterial infection, mice were infused (2 µL/g) with an enrofloxacin/saline solution (22.7 mg/kg) immediately before the heparin flush at the end of each session. Fentanyl citrate (CAS Registry Number: 990-73-8) was obtained from the NIDA Drug Supply Program. Fentanyl doses were calculated as the salt. All drugs were dissolved in 0.9% USP sterile saline. All solutions were filtered through 0.22 µm syringe filters. At the conclusion of the study, catheters were tested for patency with an infusion (2 µL/g) of a methohexital/saline solution (5 mg/kg). Rapid loss of muscle tone was interpreted as an indication of patency.

#### Acquisition

During the acquisition stage, each session began with the illumination of the house light and extension of the two response levers. When the active lever was pressed, a fentanyl infusion was delivered (56 µg/kg/infusion) and the stimulus light above the active lever was illuminated for five seconds. To reduce the probability of fentanyl overdose, a 25-s timeout immediately followed an infusion. During the timeout, the house light was off and active lever presses were recorded but had no consequences. Throughout the session, inactive lever presses were recorded but had no consequences. Mice were tested on the acquisition stage until they completed eight sessions during which at least one infusion was self-administered; these eight sessions were not required to be consecutive. Consequently, before advancing to the dose–response stage, mice were exposed to at least eight pairings of an active lever press followed by a fentanyl infusion and had received at least 448 µg/kg of intravenously self-administered fentanyl.

#### Dose–response

Following the acquisition stage, mice were tested on an additional five fentanyl doses in order to generate a dose–response curve. Doses were presented in the following order: 18, 5.6, 1.8, 0.56, 0.18 µg/kg/infusion. Mice were tested for three sessions on each dose. With these exceptions, the dose–response stage was identical to the acquisition stage.

#### Restabilization

Following completion of the dose–response curve, mice were again tested on the 56 µg/kg/infusion dose used during the acquisition stage. Mice were tested for three sessions on the restabilization stage. All other methodological details were identical to the acquisition stage and dose–response stage. The restabilization stage was used both to restabilize mice at a high dose prior to extinction and to calculate escalation of fentanyl self-administration across the dose–response stage. The difference between the final three sessions on the acquisition stage and the three sessions on the restabilization stage was used as a measure of escalation of intravenous fentanyl self-administration.

#### Extinction

Following the restabilization stage, mice were tested for seven sessions using extinction criteria. During extinction sessions, the house light was on, active and inactive levers were extended, lever presses were recorded, and mice were connected to the tether. However, drug-paired visual stimuli were not delivered during extinction sessions and the infusion pump was not activated.

#### Reinstatement: cue-induced

Following the extinction stage, mice were tested for a single session of cue-induced reinstatement. During the cue-induced reinstatement stage, methodological details were identical to the acquisition, dose–response, and restabilization stages with the exception that infusions were not delivered following an active lever press (i.e., mice were connected to the tether, the infusion pump was activated following an active lever press, but no syringe was in the infusion pump so an infusion was not delivered). Consequently, following an active lever press, mice were exposed to all visual and auditory cues that had previously been paired with a fentanyl infusion including illumination of the stimulus light above the active lever, extinguishing the house light during the timeout, and activation of the infusion pump. This design allowed for the isolation of the effect of drug-paired cues on lever pressing.

#### Reinstatement: stress-induced

Following the single cue-induced reinstatement session, mice were tested on three extinction sessions using methodological details identical to the extinction stage. Following these extinction sessions, mice were tested for a single session of restraint stress-induced reinstatement^27^. Mice were restrained for 60 min in a commercial restraining device (Tailveiner Restrainer for Mice, TV-150 STD; Braintree Scientific, Braintree, MA) immediately prior to operant testing. Following restraint, mice were tested for 60 min under conditions identical to the extinction stage. Thus, the sole difference between the extinction sessions immediately preceding the stress-induced reinstatement session was the introduction of restraint stress. This design allowed for the isolation of the effect of restraint stress on lever pressing.

#### Reinstatement: IV fentanyl-induced

Following the single stress-induced reinstatement session, mice were tested on three extinction sessions using methodological details identical to the extinction stage. Following these extinction sessions, mice were tested for a single session of fentanyl-induced reinstatement. During the fentanyl-induced reinstatement stage, mice were connected to the tether and a 56 µg/kg/infusion syringe was loaded into the syringe pump. The operant conditioning program automatically delivered 5 infusions (280 µg/kg bolus infusion) to the mouse at the beginning of the session prior to lever extension; drug-paired stimuli were not delivered. Following this, neither infusions nor drug-paired stimuli were delivered during the session; all lever presses were recorded. Thus, the sole difference between the extinction sessions immediately preceding the fentanyl-induced reinstatement session was the fentanyl infusion at the beginning of the session. This design allowed for the isolation of the effect of non-contingent IV fentanyl exposure on lever pressing.

### Statistical methods

To assess the effects of the independent variables on the dependent variables, we used analysis of variance (ANOVA). SPSS version 27 was used for all analyses. Dependent variables were lever presses and infusions; attempted infusions were used rather than infusion on extinction and reinstatement stages because an infusion was not delivered on those stages. Independent variables were dose, lever, session, stage, strain, and sex; the specific independent variables used in each analysis varied based on the hypothesis being tested and the dependent variable being assessed. Dose, lever, session, and stage were within-subjects variables. Strain and sex were between-subjects variables. The normality of dependent variables was assessed using normal probability plots. To assess homogeneity of variance, we used Mauchly’s test of sphericity. If a violation of homogeneity of variance was detected, we addressed this violation using the Huynh–Feldt correction. Post hoc tests were performed using Fisher's Least Significant Difference procedure. Broad-sense heritability was defined as the proportion of variance accounted for by strain relative to total variance. Variance components were estimated using VARCOMP in SPSS.

## Results

### Acquisition

**Figure 1:**
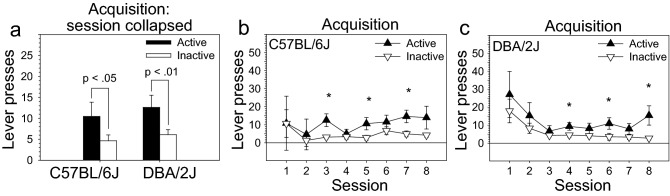
Acquisition of intravenous fentanyl self-administration in C57BL/6J and DBA/2J mice. Mice from both the C57BL/6J and DBA/2J strains pressed the active lever significantly more than the inactive lever on the acquisition stage. There were no effects of strain or sex. Data are presented as the mean ± S.E.M. *p < .05.

Following surgical recovery, mice were tested on the fentanyl IVSA acquisition stage. Mice reached criterion on the acquisition stage in 8 – 18 sessions (M = 8.56, SD = 1.95). All but five mice (84%) met this criterion on session eight, and there was no significant effect of strain or sex on sessions to meet the acquisition criterion. ANOVA revealed a main effect of lever [F (1, 28) = 14.09, *p* < .001], but not strain or sex, on lever pressing. Post hoc tests revealed that mice from both the C57BL/6J and DBA/2J strains pressed the active lever significantly more than the inactive lever on the acquisition stage (Fig. 1a–c). Neither strain nor sex effects were observed in post hoc tests (Figure S1a, S1b, S1c, S1d).

### Dose–response

**Figure 2:**
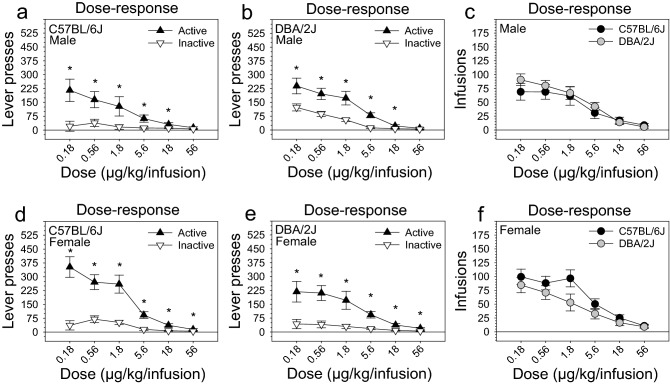
Dose–response of intravenous fentanyl self-administration in C57BL/6J and DBA/2J mice. (**a**, **b**, **d**, **e**) Male and female mice from both strains pressed the active lever significantly more than the inactive lever on almost all doses, and active lever pressing increased as fentanyl dose decreased (lowest dose vs highest dose: *p* < .001). (**c**, **f**) Number of infusions did not differ as a function of strain or sex. Data are presented as the mean ± S.E.M. *p < .05.

Following the acquisition stage, mice were tested on a dose–response stage during which fentanyl IVSA data were collected on five additional doses. The interaction of dose and lever [F (5, 140) = 32.65, *p* < .001] significantly influenced the number of lever presses on the fentanyl dose–response curve (Fig. 2a,b,d,e). The main effect of dose [F (5, 140) = 57.54, *p* < .001] and the main effect of lever [F (1, 28) = 84.38, *p* < .001] were also significant. Post hoc tests revealed that mice pressed the active lever significantly more than the inactive lever (*p* < .001; 81.19% active lever preference), and that active lever pressing increased as fentanyl dose decreased (lowest dose vs highest dose: *p* < .001). These relationships were significantly influenced by strain and sex [Dose x Strain x Sex: F (5, 140) = 3.25, *p* < .05]. Specifically, male DBA/2J mice pressed the inactive lever (Figure S2b), but not active lever (Figure S2a), significantly more than male C57BL/6J mice. This strain difference was not observed in female mice (Figure S2c, S2d). Despite significant effects of strain and sex on lever pressing, the number of infusions on the dose–response stage was influenced solely by dose [F (5, 140) = 82.30, *p* < .001]; this indicates that the number of infusions did not differ significantly as a function of strain or sex (Fig. 2c,f), and that fentanyl exposure was equivalent among groups as they entered the restabilization stage.

### Escalation

**Figure 3:**
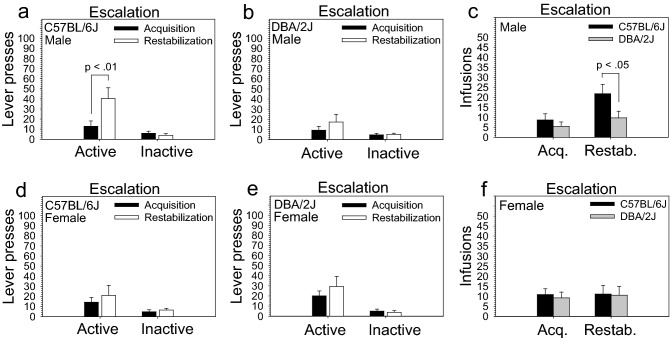
Escalation of intravenous fentanyl self-administration in C57BL/6J and DBA/2J mice. To calculate escalation, we compared performance on the acquisition stage with performance on the restabilization stage that immediately followed the dose–response curve; both stages used the 56 µg/kg/infusion dose. (**a**, **b**, **d**, **e**) Male C57BL/6J mice, but not other subgroups, significantly escalated active lever pressing (*p* < .01), but not inactive lever pressing, on the restabilization stage relative to the acquisition stage. (**c**, **f**) Moreover, male C57BL/6J mice intravenously self-administered significantly more fentanyl on the restabilization stage (*p* < .05), but not acquisition stage, relative to male DBA/2J mice; this difference was not observed in females. Data are presented as the mean ± S.E.M.

Following the dose–response stage, mice were tested on a restabilization stage to ensure that all mice were responding for a reinforcing fentanyl dose prior to entering the extinction stage. During the restabilization stage, mice were tested for three sessions on the same dose used during the acquisition stage (56 µg/kg/infusion). To determine if escalation occurred between the acquisition stage and the restabilization stage, we included stage as an independent variable in the ANOVA. ANOVA revealed a significant effect of stage on infusions [F (1, 28) = 6.55, *p* < .05] and a significant stage x lever interaction influencing lever pressing [F (1, 28) = 9.30, *p* < .01]. Post hoc tests revealed significantly greater infusions (*p* < .05) and active lever pressing (*p* < .01), but not inactive lever pressing, on the restabilization stage relative to the acquisition stage. A significant two-way stage x sex interaction influencing infusions [F (1, 28) = 4.56, *p* < .05] and a marginally significant three-way strain x lever x sex interaction influencing lever pressing [F (1, 28) = 3.79, *p* = .06] indicated that escalation was sex dependent and that lever pressing varied as a function of the relationship between strain, lever, and sex. Post hoc tests indicated that male C57BL/6J mice (Fig. 3a), but not other subgroups (Fig. 3b,d,e), significantly escalated active lever pressing (*p* < .01), but not inactive lever pressing, on the restabilization stage relative to the acquisition stage. Moreover, male C57BL/6J mice intravenously self-administered significantly more fentanyl on the restabilization stage (*p* < .05), but not acquisition stage, relative to male DBA/2J mice (Fig. 3c); this difference approached statistical significance for active lever presses (*p* = .08) (Figure S3a) but not inactive lever presses (Figure S3b). These differences were not observed in females (Figs. 3f, S3c, S3d).

### Extinction

**Figure 4:**
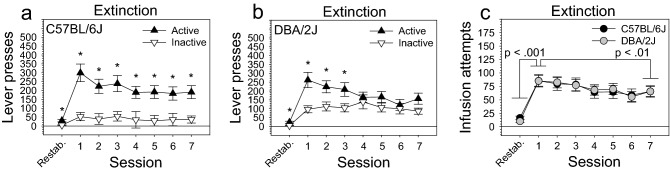
Extinction of intravenous fentanyl self-administration in C57BL/6J and DBA/2J mice. Following the restabilization stage, mice were tested for seven sessions on an extinction stage during which lever pressing had no consequences. (**a**, **b**, **c**) Mice exhibited both an extinction burst and extinction of the previously learned operant response. First, mice significantly increased active lever pressing (*p* < .001) and infusion attempts (*p* < .001) on the first extinction session relative to the restabilization stage (i.e., extinction burst). Second, mice significantly reduced infusion attempts (*p* < .01) and active lever pressing (*p* < .001), but not inactive lever pressing, on the final extinction session relative to the first extinction session (i.e., extinction of the previously learned response). These phenomena were not influenced by strain or sex. Data are presented as the mean ± S.E.M. *p < .05.

Following the restabilization stage, mice were tested for seven sessions on an extinction stage during which lever pressing had no consequences. Using lever presses as the dependent variable, ANOVA revealed a significant two-way lever x session interaction [F (7, 196) = 8.76, *p* < .001] and significant main effects of lever [F (1, 28) = 23.57, *p* < .001] and session [F (7, 196) = 20.92, *p* < .001]. Using infusion attempts as the dependent variable, ANOVA revealed a significant main effect of session [F (7, 196) = 29.95, *p* < .001]. Post hoc tests revealed that mice significantly increased active lever pressing (*p* < .001) and infusion attempts (*p* < .001) on the first extinction session relative to the restabilization stage (i.e., extinction burst) (Fig. 4a–c). Further, post hoc tests revealed that mice significantly reduced infusion attempts (*p* < .01) and active lever pressing (*p* < .001), but not inactive lever pressing, on the final extinction session relative to the first extinction session (Fig. 4a–c) (i.e., extinction of the previously learned lever pressing response). ANOVA did not reveal significant effects of strain, sex, or their interaction term. However, post hoc tests indicated that male DBA/2J mice emitted significantly more inactive lever presses than male C57BL/6J mice (Figure S4b); no other strain differences were observed for males or females (Figure S4a, S4c, S4d).

### Reinstatement: cue-induced

**Figure 5:**
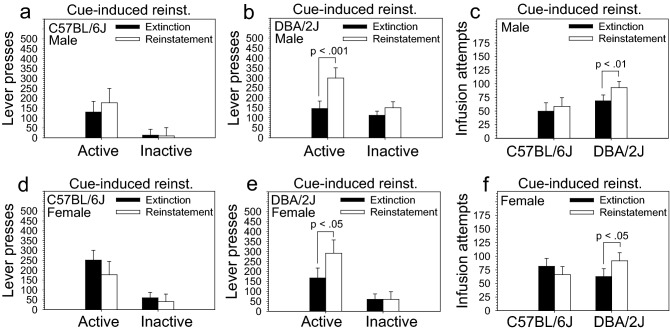
Cue-induced reinstatement of intravenous fentanyl self-administration in C57BL/6J and DBA/2J mice. Following the extinction stage, mice were evaluated on cue-induced reinstatement. Performance on a single reinstatement session was compared to performance on the extinction session from the prior day. (**a**, **b**, **c**) Male and (**d**, **e**, **f**) female DBA/2J mice, but not C57BL/6J mice, significantly escalated infusion attempts and active lever pressing, but not inactive lever pressing, on the cue-induced reinstatement stage. Cue-induced reinstatement was not influenced by sex. Data are presented as the mean ± S.E.M.

Following the extinction stage, mice were tested for a single session on cue-induced reinstatement. To determine if the reintroduction of drug-paired cues strain- or sex-dependently influenced lever pressing or infusion attempts, we compared performance on the single session of cue-induced reinstatement to performance on the extinction session from the previous day. ANOVA revealed a significant two-way interaction of stage and lever on lever pressing [F (1, 28) = 6.67, *p* < .05]. This relationship was influenced by strain as indicated by a significant three-way interaction of strain, stage, and lever on lever pressing [F (1, 28) = 7.07, *p* < .05] and a significant two-way interaction of strain and stage on infusion attempts [F (1, 28) = 7.23, *p* < .05]. Sex did not influence these relationships. Post hoc tests revealed that male and female DBA/2J mice exhibited a robust and significant increase in infusion attempts (Fig. 5c,f) and active lever pressing (Fig. 5b,e), but not inactive lever pressing, on the cue-induced reinstatement stage relative to the extinction stage. Neither male nor female C57BL/6J mice exhibited an increase in lever pressing (Fig. 5a,d) or infusion attempts (Fig. 5c,f) across these stages. ANCOVA revealed that, after adjusting for performance on the prior extinction session, the number of infusions [F (1, 27) = 7.98, *p* < .01] and active lever presses [F (1, 27) = 8.02, *p* < .01], but not inactive lever presses, on the cue-induced reinstatement stage were significantly higher in DBA/2J mice relative to C57BL/6J mice (Figure S5a, S5b, S5c); this effect was not significantly sex dependent. Heritability estimates calculated using adjusted values generated from the ANCOVA were 0.367 for attempted infusions and 0.353 for active lever presses (sex collapsed for both calculations). Heritability estimates calculated using raw values from the cue-induced reinstatement stage were 0.196 for attempted infusions and 0.150 for active lever presses.

### Reinstatement: restraint stress-induced

**Figure 6:**
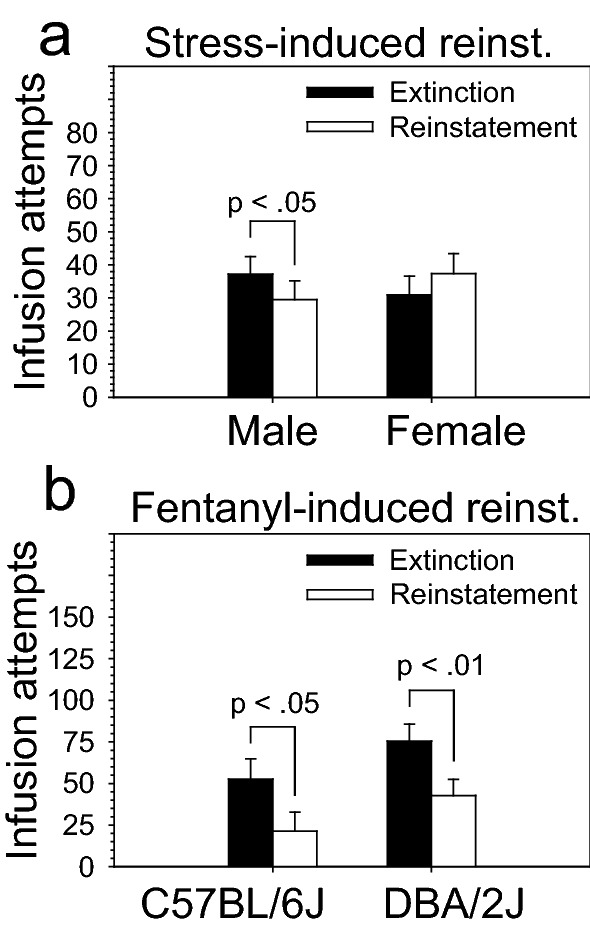
Stress-induced and IV fentanyl-induced reinstatement of intravenous fentanyl self-administration in C57BL/6J and DBA/2J mice. Mice were evaluated on restraint stress-induced and IV fentanyl-induced reinstatement. For both types of reinstatement, performance on a single reinstatement session was compared to an extinction session from the prior day. (**a**) Males, but not females, significantly *decreased* infusion attempts following acute restraint stress (*p* < .05). Notably, there was a non-significant trend in females to *increase* infusion attempts following acute restraint stress. ANOVA did not reveal a significant effect of strain on restraint stress-induced reinstatement. (**b**) As a group, mice significantly decreased infusion attempts following non-contingent IV fentanyl infusion (280 µg/kg bolus infusion). This effect was evident in both strains and was not significantly influenced by strain or sex. Data are presented as the mean ± S.E.M.

Following the cue-induced reinstatement stage, mice were tested for three extinction sessions followed by a single session on restraint stress-induced reinstatement. To determine if acute restraint stress strain- or sex-dependently influenced lever pressing or infusion attempts, we compared performance on the single session of restraint stress-induced reinstatement to performance on the extinction session from the previous day. ANOVA revealed a significant two-way interaction of stage and sex on infusion attempts [F (1, 28) = 7.21, *p* < .05]. Post hoc tests revealed that this effect was driven by a significant *decrease* in infusion attempts by males, but not females, following acute restraint stress (*p* < .05) (Fig. 6a). The interaction was further driven by an *increase* in infusion attempts by females following acute restraint stress, although the increase in females did not reach statistical significance (*p* = .10). ANOVA did not reveal significant effects of strain. However, post hoc tests indicated that male DBA/2J mice emitted significantly more inactive lever presses relative to male C57BL/6J mice on both extinction and reinstatement stages (Figure S6b); no other statistically significant strain differences were detected by the post hoc tests (Figure S6a, S6c, S6d, S6e, S6f.).

### Reinstatement: IV fentanyl-induced

Following the restraint stress-induced reinstatement stage, mice were tested for three extinction sessions followed by a single session on fentanyl-induced reinstatement. To determine if non-contingent fentanyl (280 µg/kg bolus infusion) immediately prior to testing strain- or sex-dependently influenced lever pressing or infusion attempts, we compared performance on the single session of fentanyl-induced reinstatement to performance on the extinction session from the previous day. ANOVA revealed a significant effect of stage on lever pressing [F (1, 28) = 9.08, *p* < .01] and infusion attempts [F (1, 28) = 17.59, *p* < .001]. Post hoc tests indicated that mice as a group significantly reduced lever pressing (*p* < .01) and infusion attempts (*p* < .001) on the fentanyl-induced reinstatement stage relative to the prior extinction stage. This effect was evident in both strains (Fig. 6b). In addition to the effects of stage, we observed a statistically significant interaction of strain and sex on lever pressing [F (1, 28) = 6.99, *p* < .05] and a marginally significant interaction of strain and sex on infusions [F (1, 28) = 3.97, *p* = .06]. These interactions were driven by relatively higher lever presses and infusions in male DBA/2J mice relative to male C57BL/6J mice (Figure S7a, S7b, S7c) on both extinction and reinstatement sessions; these relationships were not observed in female mice (Figure S7d, S7e, S7f). Importantly, neither strain nor sex interacted with stage.

## Discussion

### Summary

In the present study, we used intravenous fentanyl self-administration to quantify classical self-administration phenotypes and measures of addiction-like behavior in male and female mice from the founder strains of the BXD recombinant inbred mouse panel. Our primary goal was to identify strain and sex effects influencing intravenous fentanyl self-administration phenotypes and to determine if the BXD panel could be used to identify genetic mechanisms underlying variation on these phenotypes. Using data generated from these experiments, we reached three primary conclusions. First, mice from all groups acquired intravenous fentanyl self-administration (Fig. 1), exhibited a preference for the active lever and a dose–response curve (Fig. 2), and exhibited an extinction burst and extinction of the learned self-administration response (Fig. 4). Second, fentanyl intake was equivalent among groups on the acquisition stage (Fig. 1) and dose–response stage (Fig. 2); fentanyl seeking during extinction was also equivalent among groups (Fig. 4). Finally, strain effects, sex effects, or both were identified for several addiction-like behaviors (cue-induced reinstatement, stress-induced reinstatement, and escalation of intravenous fentanyl self-administration) (Figs. 3, 5, 6a). These data reveal novel strain- and sex-dependent effects on intravenous fentanyl self-administration in mice and indicate that the full BXD recombinant inbred mouse panel can be used to reveal and dissect the genetic mechanisms underlying these observed effects.

### Viability of intravenous fentanyl self-administration in the mouse

Drinking and vapor self-administration of fentanyl in mice^28–31^, intravenous self-administration of remifentanil in mice^32–35^, and intravenous self-administration of fentanyl in rats^13,36–38^ have all been described in the literature. Together, these studies have begun to uncover the biological mechanisms driving fentanyl addiction. However, intravenous fentanyl self-administration is rarely studied in mice. Therefore, using data from the present study, it is appropriate to examine main effects and interactions of experimental variables such as lever, dose, and stage to determine if intravenous fentanyl is indeed rewarding in mice and if mice can effectively regulate fentanyl taking using the intravenous route. In this regard, several patterns in the data are notable: During the acquisition and dose–response stages, mice as a group rapidly acquired the lever pressing response, exhibited a significant and robust preference for the active lever, and exhibited a significant and clear dose–response. Moreover, mice exhibited an extinction burst and reduced active lever pressing, but not inactive lever pressing, across the extinction stage. Collectively, these data indicate that intravenous fentanyl was rewarding in the tested mouse strains, that the mice were able to learn the operant response, and that the mice were able to regulate fentanyl self-administration by increasing or decreasing active lever pressing to adjust for changes in dose or operant schedule.

### Cue-induced reinstatement of intravenous fentanyl self-administration

In the present study, DBA/2J mice exhibited robust cue-induced reinstatement following extinction of intravenous fentanyl self-administration; this effect was completely absent in C57BL/6J mice (Fig. 5). Post hoc tests revealed that significant cue-induced reinstatement was observed in both male and female DBA/2J mice and was absent in both male and female C57BL/6J mice. There was no effect of sex. These findings reveal a strain- but not sex-dependent effect of drug-paired stimuli on fentanyl seeking in the founder strains of the BXD recombinant inbred panel.

Several findings from the present study suggest that the observed strain effect on cue-induced reinstatement was a primary rather than a secondary effect. First, strain differences in prior fentanyl intake are unlikely to have driven this effect: there were no strain effects on fentanyl intake during the acquisition stage or dose–response stage; strain differences during the restabilization stage were observed only in male C57BL/6J mice. Second, because fentanyl seeking was equivalent in C57BL/6J and DBA/2J mice during extinction, strain differences in fentanyl seeking itself are unlikely to have caused this effect.

It is possible that strain dependent variation in reinforcement from the drug-paired stimuli themselves may have caused, in whole or in part, the strain dependent effect observed at the cue-induced reinstatement stage. Specifically, sensory stimuli are naturally reinforcing in mice, and we have shown that DBA/2J mice self-administer significantly more sensory stimuli than C57BL/6J mice^39,40^. However, it’s important to consider that the drug-paired stimuli used in the present study were much less complex than those commonly used for sensory stimulus self-administration including those used in our own previous studies of C57BL/6J and DBA/2J mice. This is relevant because reducing the complexity of sensory stimuli reduces, and eventually eliminates, their reinforcement value in mice^40,41^. Collectively, data from the present study and previous studies suggest that genetic differences between C57BL/6J and DBA/2J mice underlie differences in the influence of drug-paired cues over fentanyl seeking following extinction of intravenous fentanyl self-administration.

### Escalation of intravenous fentanyl self-administration

From the acquisition stage to the restabilization stage, male C57BL/6J mice, but not other groups, significantly escalated active lever pressing (Fig. 3). Consequently, male C57BL/6J mice intravenously self-administered significantly more fentanyl on the restabilization stage (*p* < .05), but not acquisition stage, relative to male DBA/2J mice. These strain differences were not seen in females, and inactive lever pressing did not change significantly in any of the groups. The absence of a change in inactive lever pressing indicates that the increased intravenous fentanyl self-administration was goal-directed rather than secondary to an increase in general activity. These data indicate that goal-directed escalation of intravenous fentanyl self-administration can be indexed in the mouse, and that strain and sex influence this phenomenon. In this regard, indexing this phenotype in preclinical studies is critical for identification of the genetic drivers of addiction because escalation of drug taking is a core feature of addiction in humans^42^.

### Experimental design considerations

Several aspects of the experimental design used in the present study are worth highlighting. These include the use of a timeout between infusions, the order of dose presentation on the dose–response curve, the use of a constant number of extinction sessions rather than a performance criterion, the characteristics of the specific restrainer used for stress-induced reinstatement, and the dose of the bolus infusion on IV fentanyl-induced reinstatement.

#### Timeout following fentanyl infusions

Because of the high relevance of fentanyl overdose in human addiction, intravenous fentanyl self-administration to overdose would be an informative endpoint in mouse models of addiction. However, for several reasons, we chose to use a 25-s timeout to reduce the probability of fentanyl overdose in the present study. First, our goal was to quantify multiple measures including full dose–response curve, escalation, extinction, and reinstatement. We reasoned that without the timeout to reduce the probability of overdose, attrition would be high and our sample size for many of these measures would be insufficient. Second, we were concerned about the possibility of a selection bias. Specifically, if the mice that were most prone to exhibiting addiction-like behaviors overdosed during acquisition, those mice would not be included in subsequent measures. Finally, we reasoned that the total active lever presses variable and total infusions variable would be positively predictive of self-administration to overdose.

#### Dose presentation on the dose–response curve

In the present study, we held dose presentation constant on the dose–response curve rather than using a Latin square design. Specifically, all mice began testing with the highest dose and ended with the lowest dose before being restabilized prior to extinction. One consequence of this high-to-low presentation is that strain differences in opioid tolerance^43^ could influence performance on later doses, although strain differences in tolerance may influence performance on later doses even when using a Latin square design. Benefits to holding dose presentation constant include low standard deviation at each dose, reduced chance of confounding strain and dose-presentation if attrition occurs, and freedom to use any subgroup sample size without being constrained by the number of dose combinations in the Latin square. This final point is especially important in QTL mapping studies in which maximum statistical power is often achieved with a large number of strains and a relatively small subgroup sample size^44,45^.

#### Extinction criteria

In the present study, we tested all mice for seven sessions on the extinction stage before advancing them to the cue-induced reinstatement stage. Another viable option would have been to advance mice only after they had achieved a predetermined extinction criterion. For example, some investigators choose to continue extinction trials until the level of extinction responding is equal to the pre-extinction baseline. We chose to hold extinction sessions constant because the present study was intended to serve as proof of principle for a large systems genetics study using all strains from the BXD recombinant inbred panel. In this regard, mice from recombinant inbred panels exhibit massive phenotypic variation, and many strains will never fully extinguish responding on operant conditioning paradigms. Therefore, in order to avoid attrition consequent to failure to meet a criterion, our strategy with recombinant inbred panels is to capture phenotypic variation over a predetermined number of sessions and then harness that variation to identify causal genetic variants. Although this strategy enables efficient identification of causal variants, it does not enable complete characterization of the extinction curve. For example, in the present study, data suggest that active lever responding is not fully extinguished (i.e., the rate of responding on extinction sessions is higher than during restabilization). Following QTL mapping, these issues can be addressed in gene candidate validation studies by adjusting the experimental design in order to fully characterize behavioral phenomena such as the extinction curve.

#### Effect of restrainer on stress-induced reinstatement

In the present study, we identified a sex-dependent effect of restraint stress on fentanyl seeking. Specifically, male mice significantly reduced fentanyl seeking following restraint stress, whereas female mice did not (Fig. 6a). Indeed, female mice increased fentanyl seeking following restraint stress although this effect did not reach statistical significance. Collectively, these data suggest that sex may influence restraint stress-induced reinstatement in mice from the C57BL/6J and DBA/2J strains. In this regard, it is important to consider that the duration of restraint and the choice of restrainer may have influenced our findings: First, the sex dependent effect of stress on reinstatement may have been caused by the smaller size of female mice relative to males. Specifically, because we used the same size polycarbonate restrainer for both males and females, the stress experienced by males may have been different from that experienced by females. Second, the effect of acute stress on fentanyl seeking may be dependent on the duration of that stress. Consequently, the effects that we observed may have been unique to the duration of acute restraint stress that we used. In future studies, using duration of restraint stress as an independent variable may reveal main effects of strain or interactions of strain and sex that were not detected here.

#### Bolus infusion dose on IV fentanyl-induced reinstatement

Although we hypothesized that mice would increase fentanyl seeking during IV fentanyl-induced reinstatement, mice actually decreased fentanyl seeking relative to the previous extinction session (Fig. 6b). One explanation for this is that a bolus dose of 280 µg/kg is too high for IV fentanyl-induced reinstatement in the C57BL/6J and DBA/2J strains. This hypothesis is consistent with the relatively low number of infusions on the 56 µg/kg/infusion dose during acquisition and restabilization. These data suggest that, in future studies, a lower dose should be used for IV fentanyl-induced reinstatement in mice. Moreover, including dose as an independent variable on the IV fentanyl-induced reinstatement stage may reveal strain-dependent effects that were undetected here.

### Systems genetics using recombinant inbred mouse panels for discovery of the genetic mechanisms underlying the observed strain- and sex-dependent effects on intravenous fentanyl self-administration

The present study indicates that intravenous fentanyl self-administration can be quantified in mice, and that strain and sex influence addiction-like fentanyl seeking in C57BL/6J and DBA/2J mice. These data indicate that the full BXD recombinant inbred mouse panel can be used to identify the genetic mechanisms driving variation on these phenotypes. Based on the data presented here, the most viable target for genetic dissection in the BXD is cue-induced reinstatement following intravenous fentanyl self-administration. Identifying the genetic mechanisms driving this phenotype is critical for understanding compulsive fentanyl seeking because environmental cues play a strong role in drug cravings^46^. Escalation of intravenous fentanyl self-administration may also be a viable target in the BXD. To dissect the underlying mechanisms of these phenotypes using a systems genetics approach, mice from many BXD strains would be phenotyped on intravenous fentanyl self-administration followed by extinction and cue-induced reinstatement. These behavioral data, along with gene expression data from fentanyl exposed and drug naïve mice, would be integrated with publicly available genotype data and used in a comprehensive systems genetics analysis^18^. Using this approach, we have previously identified genetic mechanisms influencing addiction-relevant phenotypes including intravenous cocaine self-administration^6,19,21^. Collectively, the data presented here provide a path for discovery and dissection of genetic mechanisms driving fentanyl addiction.

## Supplementary Information

Supplementary Information.

## Data Availability

The dataset used in this study is available from the corresponding author on request.
