# Supplementary material for: Intravenous fentanyl self-administration in male and female C57BL/6J and DBA/2J mice
Source: Sci Rep. 2023 Jan 16;13:799. doi: 10.1038/s41598-023-27992-8 (PMC9842734; doi:10.1038/s41598-023-27992-8)
Supplement: Supplementary file 1 — Supplementary Information. [file 41598_2023_27992_MOESM1_ESM.pdf]

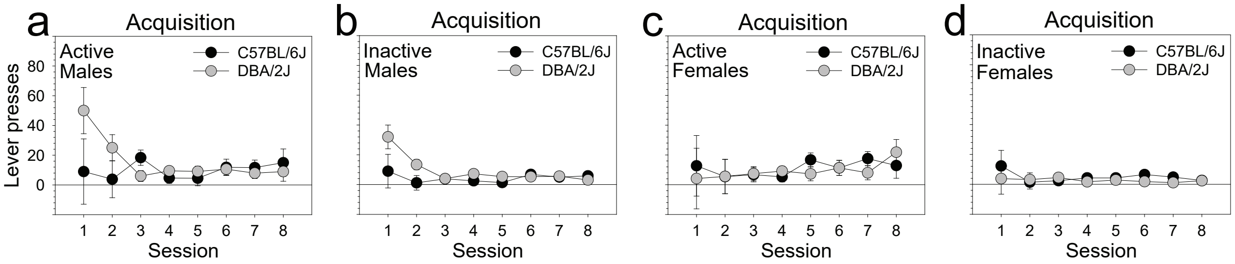

Figure S1. Acquisition of intravenous fentanyl self-administration. (a, b, c, d) Performance of C57BL/6J and DBA/2J strains did not differ significantly on the acquisition stage.

Data are presented as the mean ± S.E.M.

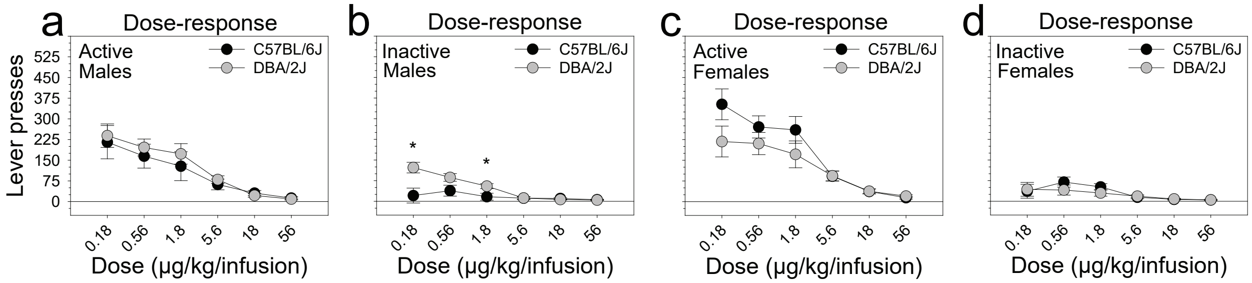

Figure S2. Intravenous fentanyl self-administration dose-reponse. (a, b, c, d) Male, but not female, DBA/2J mice pressed the inactive lever, but not active lever, significantly more than male C57BL/6J mice on two doses.

Data are presented as the mean ± S.E.M.
* $p < .05$

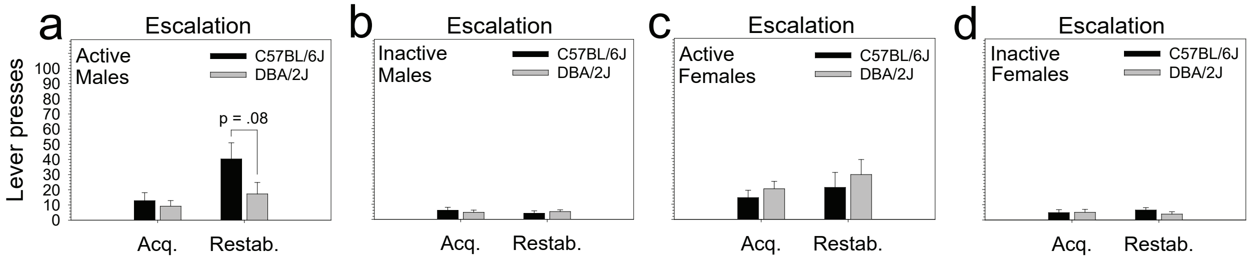

Figure S3. Escalation of intravenous fentanyl self-administration. (a, b) The difference in active lever presses, but not inactive lever presses, between male C57BL/6J mice and male DBA/2J mice on the restabilization stage approached statistical significance (p = .08).
(c, d) This strain difference was not observed in female mice.

Data are presented as the mean ± S.E.M.

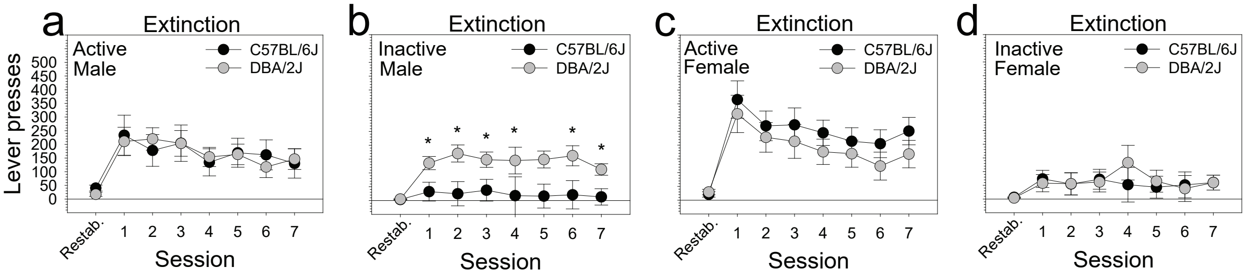

Figure S4. Extinction of intravenous fentanyl self-administration. (a, b, c, d) No significant effects of strain, sex, or their interaction term were observed in the ANOVA. However, post hoc tests indicated that male DBA/2J mice emitted significantly more inactive lever presses than male C57BL/6J mice.

Data are presented as the mean ± S.E.M.
* $p < .05$

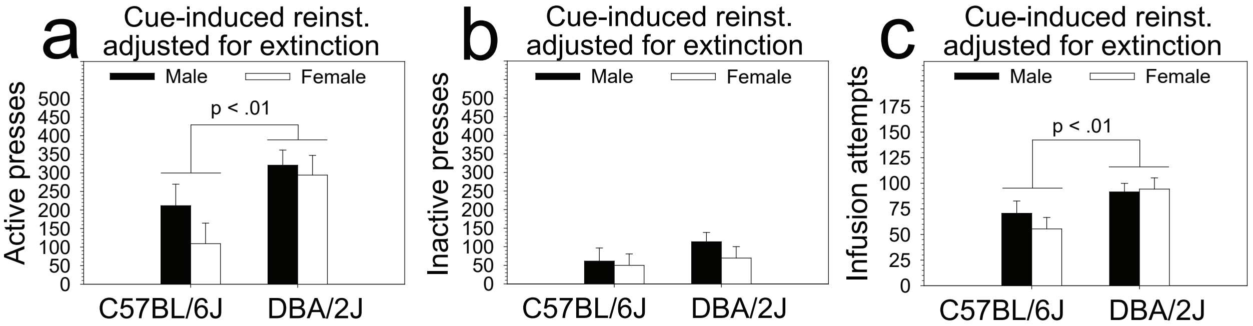

Figure S5. Cue-induced reinstatement following extinction of intravenous fentanyl self-administration. (a, b) Lever presses and **(c)** infusions on the cue-induced reinstatement session after adjusting for performance on the previous extinction session using ANCOVA. DBA/2J mice emitted significantly more active lever presses ($p < .01$), but not inactive lever presses, and intravenously self-administered more infusions ($p < .01$) relative to C57BL/6J mice. Sex did not significantly influence this effect.

Data are presented as the mean ± S.E.M.

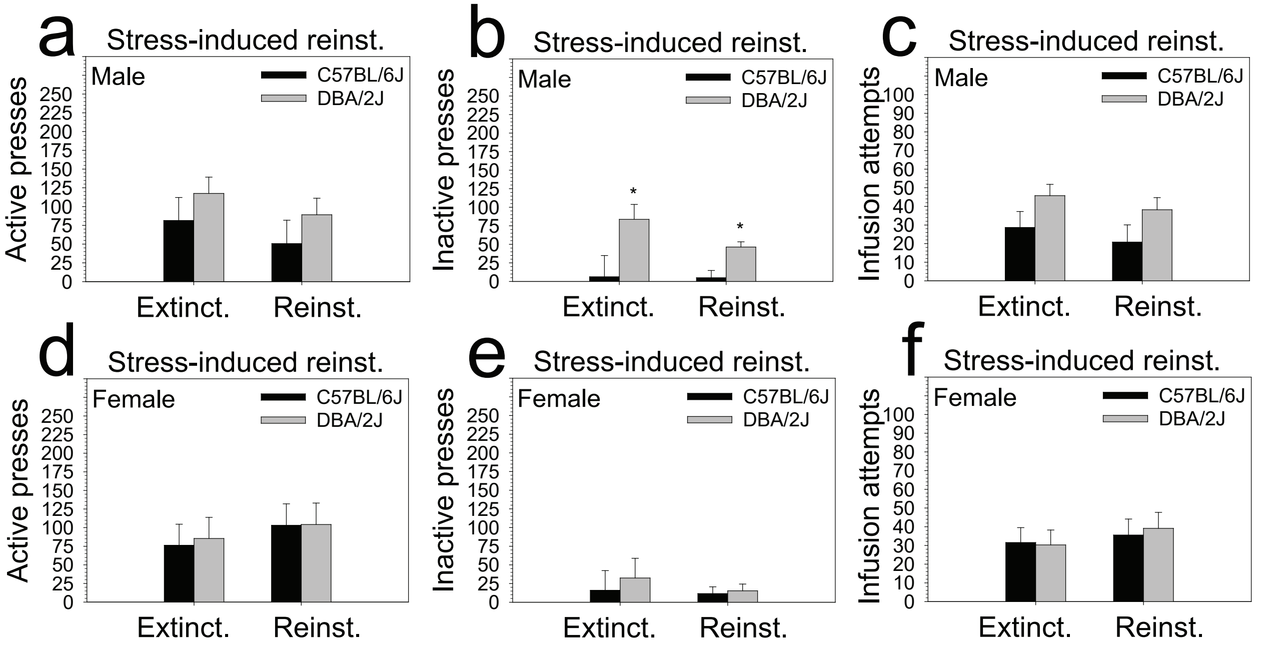

Figure S6. Stress-induced reinstatement following extinction of intravenous fentanyl self-administration. (a, b, c, d, e, f) ANOVA did not reveal significant effects of strain. However, post hoc tests indicated that male DBA/2J mice emitted significantly more inactive lever presses relative to male C57BL/6J mice on both extinction and reinstatement stages. No other statistically significant strain differences were detected.

Data are presented as the mean ± S.E.M.
* $p < .05$

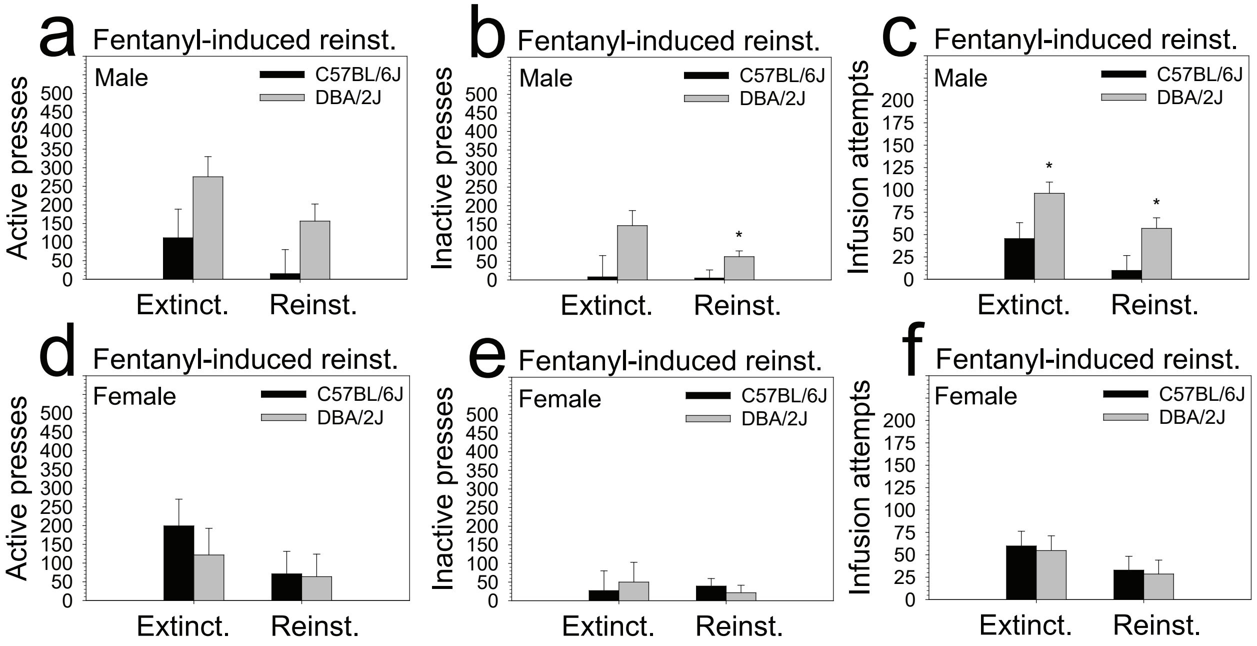

Figure S7. Fentanyl-induced reinstatement following extinction of intravenous fentanyl self-administration. (a, b, c) We observed a statistically significant interaction of strain and sex on lever pressing [$F(1, 28) = 6.99$, $p < .05$] and a marginally significant interaction of strain and sex on infusions [$F(1, 28) = 3.97$, $p = .06$]. These interactions were driven by relatively higher lever presses and infusions in male DBA/2J mice relative to male C57BL/6J mice on both extinction and reinstatement sessions. **(d, e, f)** These relationships were not observed in female mice. Importantly, neither strain nor sex interacted with stage.

Data are presented as the mean ± S.E.M.
* $p < .05$
